# Decreasing Trends in Antibiotic Consumption in Public Hospitals from 2014 to 2017 Following the Decentralization of Drug Procurement in Myanmar

**DOI:** 10.3390/tropicalmed6020057

**Published:** 2021-04-20

**Authors:** Khin Hnin Pwint, Kyaw Soe Min, Wenjing Tao, Hemant Deepak Shewade, Khin Thet Wai, Hnin Aye Kyi, Sushma Shakya, Badri Thapa, Rony Zachariah, Zaw Than Htun

**Affiliations:** 1 Department of Medical Research, Ministry of Health and Sports, Yangon 11191, Myanmar; khinthetwaidmr@gmail.com (K.T.W.); zawthanhtun@mohs.gov.mm (Z.T.H.); 2 Department of Medical Services, Ministry of Health and Sports, Naypyidaw 15015, Myanmar; dr.ksmin@gmail.com (K.S.M.); hninayekyi21@gmail.com (H.A.K.); 3 Unit for Antibiotics and Infection Control, Public Health Agency of Sweden, 171 65 Stockholm, Sweden; wenjing.tao@fohm.se; 4 International Union Against TB and Lungs Disease (The Union), 75000 Paris, France; hemantjipmer@gmail.com; 5 The Union South East Asia, New Delhi 110001, India; 6 World Health Organization, Lalitpur 44700, Kathmandu, Nepal; shakya_sushma@hotmail.com; 7 World Health Organization, 403 (A1), Bahan Township, Yangon 11201, Myanmar; THAPAB@who.int; 8 Special Program for Research and Training in Tropical Disease (TDR), 20 Avenue Appia, 1211 Geneva 27, Switzerland; zachariahr@who.int

**Keywords:** operational research, antimicrobial resistance, antibiotic stewardship, AWaRe, SORT IT, surveillance, drug monitoring, health system resilience

## Abstract

(1) Background: In 2014, drug procurement for public hospitals in Myanmar was decentralized to a pull system. This might lead to increasing trends in the consumption of broad-spectrum and last-resort antibiotics. For fiscal years 2014-2017, we assessed annual antibiotic consumption trends and patterns in total defined daily doses (DDDs). (2) Methods: We followed World Health Organization (WHO) methodology for surveillance of antimicrobial consumption based on hospital antibiotic procurement records (as a proxy). (3) Results: In 32% of all public hospitals where data were retrieved, total antibiotic consumption reduced by 19% between 2014 (7,122,852 DDD) and 2017 (5,794,904 DDD). Consumption per 1000 inhabitants per day (<200 bed hospitals) also reduced from 0.6 to 0.3. Over 60% of procurement was for beta-lactam antibiotics and quinolones; quinolones decreased over time. Consumption of first-line antibiotics increased (42% in 2014 to 54% in 2017), whereas broad-spectrum antibiotics decreased (46% in 2014 to 38% in 2017). Linezolid was the only last-resort antibiotic procured. There was a progressive reduction in per capita government current health expenditure from approximately 9.2 US$ in 2014 to 8.3 US$ in 2017. (4) Conclusions: Antibiotic consumption decreased over time in public hospitals. This first study provides a baseline for developing an antibiotic consumption surveillance system in Myanmar.

## 1. Introduction

Antimicrobial resistance (AMR) has become a recognized threat to the effective prevention and treatment of bacterial infections globally [1,2]. Importantly, in low- and middle-income countries, the potential for AMR-related mortality may be higher because of the larger burden of infectious diseases, delayed presentation with associated severe illness, limited access to laboratory diagnostics (particularly microbiology), and reduced availability of second-line antibiotics [3].

Appropriate and inappropriate antibiotic use creates a selection pressure for bacteria to select antibiotic resistance. Inappropriate use of antibiotics is one of the main drivers of the emergence and spread of AMR. This includes using antibiotics to treat conditions that are not caused by bacterial infections, the use of the wrong type or dosage of antibiotics, the wrong route of administration, or the duration of treatment. Use of prophylactic antibiotics in surgery, such as cesarean sections and gastrointestinal surgery, is common when not always justified. Almost half of all antibiotics used in human health care can be considered inappropriate [4]. There is an established link between levels of antibiotic use in humans and the emergence of AMR, implying that a reduction of irrational consumption of antibiotics could favorably limit resistance development [5,6,7]. In low- and middle-income countries, the development of antimicrobial resistance has been linked with poor antibiotic quality, easy access, misuse, and inadequate AMR surveillance. A situation analysis from South East Asian countries, including Myanmar, has shown high antibiotic use and poor implementation of policies to encourage appropriate use [8,9]. Myanmar is conducting antimicrobial resistance surveillance through its WHO Global AMR Surveillance sentinel sites and public hospitals [10]. AMR data from 26 public hospitals in 2017 showed high levels of resistance. Multidrug-resistant isolates have been reported, including *Escherichia coli* (83%), *Klebsiella pneumoniae* (61%), *Pseudomonas species* (30%), methicillin-resistant *Staphylococcus aureus* (10%), extended spectrum beta-lactamase (ESBL) producing *Escherichia coli* (34%), *Klebsiella pneumoniae* (32%), carbapenem-resistant *Acinetobacter* spps. (44%), and *Pseudomonas* spps. (32%) [11].

To improve access and, at the same time, preserve the effectiveness of existing antibiotics, particularly second-line and “last resort” antibiotics, WHO has categorized antibiotics into three categories, named AWaRe—Access, Watch, and Reserve antibiotics. The Access category includes antibiotics needed for common infections and should be available at all times, affordable, and quality-assured [9]. The Watch category includes broad-spectrum antibiotics that should be used with caution because of their high potential to develop AMR, while the Reserve category contains “last resort” antibiotics for multi-drug resistant infections [12].

One of the pillars of the World Health Organizations’ (WHO) Global Action Plan and Myanmar AMR Action Plan to tackle AMR is to optimize the use of antibiotics through antibiotic stewardship [2]. In 2014, Myanmar changed drug procurement for public hospitals from a centralized system by the Department of Medical Services to a decentralized one in which hospitals undertake their own local procurement—essentially a change from a push system to a pull system of drug procurement. This change was to increase the availability of all medicines at decentralized sites while reducing administrative and logistic loads at the central level [13].

A concern is that this policy change might be associated with increasing trends in antibiotic consumption after 2014. A shift could be to more broad-spectrum antibiotics (belonging to Watch and Reserve categories) as the demand for antibiotics could be more easily influenced by individual physician preferences and persuasive effects of pharmaceutical companies. Data on antimicrobial consumption using a standardized metric of defined daily doses (DDDs) and by AWaRe categories provide an important “handle” for countries like Myanmar to better understand the trends and amounts of antibiotics used at the national level. This could inform policies, regulations, and interventions to optimize the use of antibiotics. It could also serve as a surveillance baseline and a yardstick for future monitoring and evaluation.

Myanmar is yet to report to WHO on surveillance for antibiotic consumption at the national level and at health facilities. We thus aimed to assess antibiotic procurement (as a proxy for consumption) in three categories of hospitals: procurement for hospitals with up to 200 beds, hospitals with 200 or more beds, and central (specialist) hospitals.

The specific objectives were to assess for the fiscal years 2014–2015 to 2017–2018, (a) the overall trend in annual antibiotic consumption (in total DDDs, and for state and regional procurement, the total DDD per 1000 inhabitants per day), (b) the proportion (%) of total consumption by pharmacological subgroup, AWaRe categories, and administration route, and (c) the top ten most consumed antibiotics by hospital category.

## 2. Materials and Methods

### 2.1. Study Design

This is a cross-sectional study involving drug procurement records from hospitals in Myanmar.

### 2.2. General Setting

Myanmar lies in South East Asia and has a population of 51 million, according to the 2014 census data, with a predominantly rural population (70%). Health services are provided by the public and private sectors and non-government organizations (NGOs). The Department of Medical Services of the Ministry of Health and Sports is responsible for the procurement, storage, and distribution of medicines to all public health institutions.

### 2.3. Specific Setting—The Structure of Health Facilities

In Myanmar, health care services are largely provided by the public sector, which had 1115 public hospitals, while the private sector had 187 hospitals (17% of the total) in 2016 [13]. The public health system has a tiered structure and, from a procurement perspective, is categorized as follows: hospitals with less than 200 beds, hospitals with 200 or more beds, and central/specialist hospitals [13].

Drugs are provided free of charge in the public sector, but patients are required to purchase from private pharmacies if the prescribed drugs are out-of-stock or not included in the list of drugs procured by the hospital. There are national treatment guidelines for infectious diseases, and some central hospitals have also developed their own treatment guidelines on antibiotic use. Microbiology laboratory facilities to guide antibiotic prescriptions are available only in central, regional, and state hospitals.

### 2.4. Drug Procurement Before and After Decentralization

Drug procurement is according to the budget allocation and implemented in line with the Myanmar fiscal year (April to March). Prior to decentralization (before 2014), drugs were procured and distributed by the central medical store depot (CMSD) of the Division of Medical Care (former name of Department of Medical Services) and distributed to government health facilities by a “push” system (central level down to district/township level). The budget allocation was estimated for each health facility according to the number of beds of the facility and records of antibiotic usage. Purchase by the CMSD was about 70% from government facilities (mostly Myanmar Pharmaceutical Factory—MPF) and 30% from outside companies. During this time, the needs were underestimated, and frequent stock-outs were common. Moreover, as government budgets for generic drugs were less than 0.2 USD/capita/year, medicines supplied by the CMSD were insufficient, leading to stock-outs and resulting in patients having to purchase drugs from private pharmacies [14].

**Figure 1:**
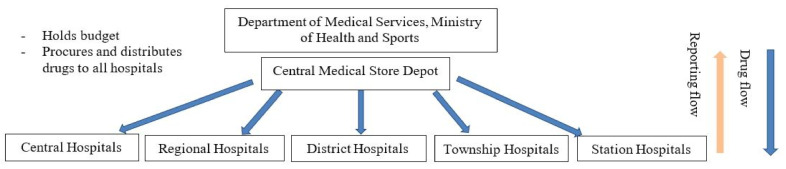
Drug procurement, distribution, and reporting before decentralization of procurement in Myanmar.

**Figure 2:**
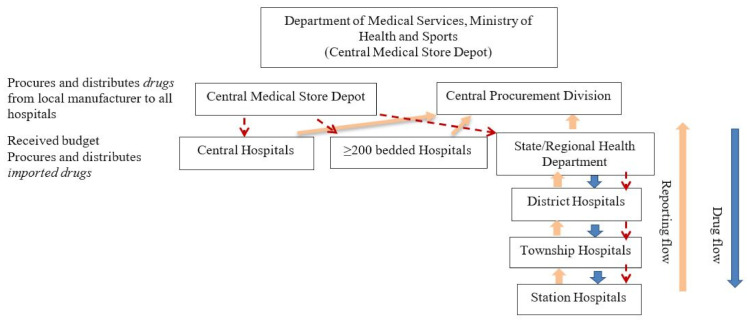
Drug procurement, distribution, and reporting after the decentralization of drug procurement in Myanmar.

Decentralization was initiated during the 2014–2015 and became fully functional in the 2015–2016 fiscal year. The decision to decentralize drug procurement was taken to alleviate the high workload related to drug procurement and related activities at the Division of Medical Care (central level). There are two ways to distribute and purchase medicines for hospitals. The first involves asking hospitals for a list of essential medicines they need. The Division of Medical Care submits this list to the Myanmar Pharmaceutical Industry (Ministry of Industry), which delivers the medicines. The second is for hospitals with 200 beds and more to be given a specific budget to purchase medicine based on need every six months. For hospitals with less than 200 beds (station, township, or district hospitals), a budget is given to their respective region or state health authorities. Each hospital has a procurement committee under the leadership of the medical superintendent (hospital director), who takes decisions on drug procurement issues. Figure 1 and Figure 2 show the drug procurement, distribution, and reporting lines prior to and after decentralization.

### 2.5. Anatomical Therapeutic Chemical (ATC)/Defined Daily Dose (DDD) Classification System

Consumption was estimated using the WHO methodology for surveillance of antimicrobial consumption and based on the ATC/DDD classification system [15]. The ATC system codes drugs according to their therapeutic, pharmacological, and chemical properties. To measure the consumption of drugs, the methodology uses the numbers of DDDs.

The DDD is the assumed average maintenance dose per day of an antimicrobial substance(s) used for its main indication in adults and is assigned to the active ingredient with an existing ATC code. To adjust for population size, antibiotic consumption is usually presented as the number of DDDs per 1000 inhabitants per day (DID). This metric can be roughly interpreted as the number of individuals per 1000 inhabitants on antibiotic treatment per day [15].

### 2.6. Study Inclusion and Periods

The study included three procurement categories of public hospitals in Myanmar for whom drug procurement data was available for the entire study period. These were hospitals with less than 200 beds in the same eight regions/states, hospitals with 200 or more beds, and central/specialist hospitals. For the estimation of consumption, we included antibiotics for systemic use, i.e., including all antibiotics categorized under the ATC group J01. Anti-tuberculosis drugs and antibiotics used for local therapy (e.g., topical creams, eye/ear drops) are not included in the WHO surveillance methodology for consumption and were thus excluded [15]. The study period included data for the period 2014 to 2017.

### 2.7. Data Variables, Sources of Data, and Statistical Analysis

Data on public hospitals and coverage populations were obtained from annual hospital reports. Details of the antibiotics procured were obtained from the drug procurement reports available from the Procurement Division, Department of Medical Services, Naypyidaw. For DDD calculation, variables collected included International Non-proprietary Name (INN), the strength of the active ingredient(s) in the unit it is specified, route of administration, and the number of units procured of the product.

Details on the procured antibiotic products were manually entered into a formatted Microsoft excel template provided by WHO with embedded macros to generate DDD per product and year. Annually aggregated DDDs were then stratified according to ATC level 3 and level 5, route of administration, and AWaRe categories.

For hospitals with less than 200 beds, antibiotic consumption in DDDs was adjusted for population size in the hospital catchment area by standardization for 1000 inhabitants per day. However, as information on the catchment area was not available for hospitals with 200 beds or more and central hospitals, DDD standardization for 1000 inhabitants was not possible. Data were managed and analyzed using Stata 15.1 (StataCorp LP, College Station, TX, USA).

## 3. Results

### 3.1. Characteristics of The Study Population (Included Hospitals)

**Figure 3:**
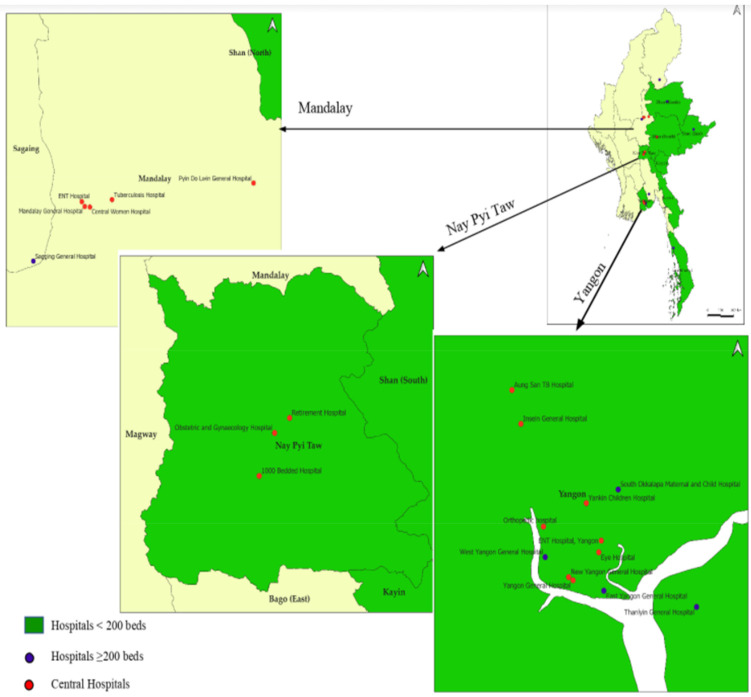
Geographic mapping of public hospitals included in the study in Myanmar (2014–2017).

The total number of public hospitals in Myanmar increased from 975 in the 2014–2015 fiscal year to 1122 in 2017–2018. Of these, an average of 32% had data on procurements for the entire study period and was included in the analysis. The included hospitals, stratified by hospital category, are shown in Table 1. Their geographic locations are shown in Figure 3.

### 3.2. Trend in Antibiotic Consumption in Total Defined Daily Doses (DDD)

The antibiotic consumption stratified by hospital categories and years is shown in Table 2. The total DDD reduced from the fiscal year 2014–15 to 2016–2017 and then increased in 2017–2018 but remained lower than the levels in the first two fiscal years (2014–2015 and 2015–16). The lowest DDD across all hospital categories occurred during 2016–17 and was most marked in hospitals with 200 or more beds.

**Figure 4:**
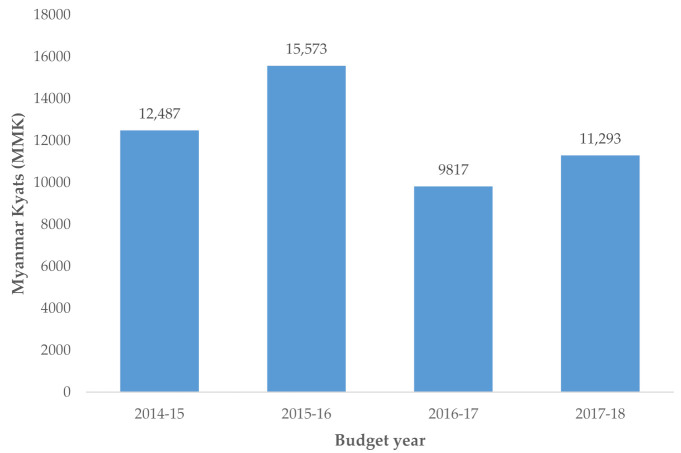
Per capita government current health expenditure in Kyats, Myanmar, 2014–2017.

In hospitals with <200 beds, the DDD by 1000 inhabitants per day also declined from 0.6 in 2014–2015 to 0.3 in 2016–2017 and remained the same thereafter. During the study period, there was a progressive reduction in per capita government current health expenditure from 12,487 Myanmar Kyats (9.2 US$) in 2014–2015 to 11,293 Myanmar kyats (8.3 US$) in 2017–2018. The fiscal year 2016–2017 had the lowest health expenditure, which was 23% lower than in 2014–2015 (Figure 4).

### 3.3. Proportion (%) of Total Antibiotic Consumption by Pharmacological Subgroup

The proportion of antibiotics consumed by the pharmacological subgroup is shown in Table 3. Beta-lactam and penicillin (J01C), other beta-lactam antibacterials (J01D), and quinolones (J01M) were the most consumed pharmacological subgroups in all categories of hospitals. The proportion of quinolones consumed seemed to decrease with years across all hospital categories, whereas the proportional consumption of beta-lactam and penicillin increased in central/specialist hospitals.

### 3.4. Antibiotic Consumption in Defined Daily Doses and Proportions by Access, Watch, and Reserve Categories

Table 4 shows the antibiotic consumption by Access, Watch, and Reserve categories. The main consumption was in the Access and Watch categories, with an increasing proportion of Access antibiotics (42% in fiscal years 2014–2015 to 54% in 2017–2018) and decreasing proportion of Watch antibiotics (46% in fiscal years 2014–2015 to 38% in 2017–2018). Reserve antibiotics were not consumed in hospitals with less than 200 beds. In the other hospital categories, consumption of Reserve antibiotics was seen only in fiscal years 2014–2015 (0.1%) and 2015–2016 (0.03%), after which there was none. Linezolid (J01XX08) was the only antibiotic in the Reserve group procured by these hospitals.

### 3.5. Antibiotic Consumption by Route of Administration

Antibiotic consumption by route of administration is shown in Table 5. In hospitals with ≥200 beds, the proportion of antibiotics used by parenteral route was high in 2015–2016 (54%) and in 2017–2018 (70%).

### 3.6. The Top Ten Most Consumed Antibiotics by Hospital Category

The top ten most consumed oral antibiotics stratified by hospital categories are shown in Table 6 and parenteral antibiotics in Table 7. There were minimal differences seen in the types of oral antibiotics between different hospital categories. The most common combinations of penicillins for which DDD could be estimated were amoxicillin or ampicillin combined with cloxacillin. Regarding parenteral antibiotics, there were considerable variations in proportions of metronidazole, amoxicillin and clavulanic acid, and ceftriaxone.

Table 8 shows the consumption of other beta-lactam antibacterials. Third-generation cephalosporins (J01DD) were the most consumed in all hospitals (range 55% to 85%) and with a rising trend in hospitals with ≥200 beds and central hospitals over the years.

## 4. Discussion

This is the first study from Myanmar showing that decentralization of drug procurement since 2014 was neither accompanied by an increasing trend in antibiotic consumption nor was a shift from Access to more broad-spectrum antibiotics belonging to the Watch and Reserve categories.

The study findings are important and suggest that introducing a locally tailored pull system of drug procurement did not perversely affect antibiotic consumption due to possible factors, such as clinicians’ preferences and/or persuasive effects of pharmaceutical companies. Importantly, this data serves as a surveillance check (a yardstick) to inform and improve antibiotic consumption monitoring in Myanmar. The experience also suggests the operational feasibility of using antibiotic procurement data for estimating national antibiotic consumptions, which can be fed into the WHO report on surveillance of antibiotic consumption. On a broader level, such operational research could help build health system resilience and accelerate efforts towards achieving sustainable development goals.

The study strengths are that we included over 30% of all public hospitals in 12 regions/states in the country; included three of the main categories of hospitals; assessed trends over four fiscal years using the same hospitals and used the WHO methodology for calculating and presenting the results [15]. Data on antibiotic consumption were cross-validated between the central procurement division and hospital reports, and we thus believe they are robust in representing the actual trends on the ground. We also adhered to the STROBE guidelines for the reporting of observational studies in epidemiology [16].

The main study limitation is that our procurement data did not cover public hospitals in all the states and regions, health facilities in the private sector, health facilities run by other ministries (like Railway), locally manufactured antibiotics, imported drugs, or drug donations received by specific hospitals. Antibiotic procurement through these channels, as well as community procurement, are aspects for future research. We also did not have pre-decentralization data to conduct a before-and-after comparison. Further, procured antibiotics may not reflect actual use, e.g., when in stock and not dispensed. Besides, when antibiotics are unavailable or out of stock, they may have to be purchased by patients, which may be particularly relevant to Watch and Reserve categories and will result in underestimations. We were also unable to assess consumption in relation to possible changes in patient numbers and infectious disease patterns over the years, which could influence antibiotics consumption. This is an area for future research, and these are important variables to be included while developing AMR surveillance systems in Myanmar. In addition, antibiotics that do not have assigned DDD were not included. This is an inherent limitation of the WHO ATC/DDD classification system. Understandably, our consumption figures are thus likely to be underestimated. However, our decision to use trend data in this study would allow such biases to be consistent over time. Finally, our analysis used a combination of levels of health care (central, regional, and district) and bed capacity (<200 beds or ≥200 beds), which are two different dimensions, and as such, there may be a limited correlation between the two.

Despite these limitations, the study has a number of policy and practice implications. First, in comparison to 2014, total antibiotic consumptions (in DDD) dropped and were lowest in the 2016–2017 fiscal year. This seems to follow the pattern of similar declines in national recurrent expenditure for health. When standardized to 1000 inhabitants per day, antibiotic consumption in hospitals with <200 beds also reduced from 0.6 to 0.3. Similar reports from other countries reported to WHO ranged from 4.4 (Burundi) to 64.4 (Mongolia); however, those figures were for national consumption data [15]. Our standardized figure of antibiotic consumption (in hospitals <200 beds) was 0.3–0.6, which seems low and might be a pointer to access and budgetary constraints. The fact that declines in the recurrent budget were closely associated with similar reductions in drug consumption adds logic to the tale. A provincial study in China evaluated antibiotic consumption patterns using procurement data in health care facilities from 2012 to 2016 after provincial centralized bidding procurement. The total antibiotic consumption in all public healthcare facilities decreased between 2012 and 2016, which is in line with our study findings [17].

Second, the quinolone group of antibiotics showed a reducing trend over the years. This is encouraging as this antibiotic, since 2017, has been classified by WHO as one of the “highest priority critically important antibiotics” that should be used prudently both in humans and animals [18]. Restricting the use of quinolones is important as they are one of the few therapies for serious *Salmonella* spp. and *Escherichia coli* infections. Further, this antibiotic class is vital for the treatment of serious urinary tract infections and for the construction of second-line drug regimens for multi-drug resistant tuberculosis (MDR-TB) in humans.

The finding that linezolid (used for tuberculosis treatment) was the only Reserve antibiotic available (even at the central hospital level) implies that antibiotics in this category are not being procured, which points to an access issue. Reserve antibiotics are expensive, and in the light of budgetary cuts, the procurement choice might have been to allocate spending to commonly used Access and Watch categories of antibiotics. The National List of Essential Medicines of Myanmar has linezolid as the only Reserve antibiotic, and this may need to be reviewed [19]. The lack of other Reserve antibiotics would imply that patients are obliged to purchase such antibiotics at their own cost in case of multi-resistant infections.

Third, in hospitals with over 200 beds, we found fluctuations in proportions of parenteral antibiotics, reaching a high of 70% in 2017–2018. This is concerning, and although we do not know the exact reasons for this finding, it merits urgent investigation. The fact that metronidazole was the most common parenteral antibiotic used in hospitals with less than 200 beds also merits investigation. This antibiotic is commonly used for surgical prophylaxis prior to and during abdominal and gynecological surgery. It is also often used to cover anaerobic bacteria in maxillofacial and dental surgery. Verifying the rationale behind this finding and whether (or not) justified would require specific quantitative and qualitative research.

With regards to cephalosporins, we observed a progressive increase in the use of third-generation cephalosporins in all hospital categories over the years. As this is a broad-spectrum antibiotic that is accessible, relatively cheap, and effective against monitoring several bacterial infections, its antibiotic resistance pattern would be useful to guide individualized treatment and empirical use.

Finally, the field experience gained from this study would provide the impetus of the need for developing a national electronic logistic management information system to capture antibiotic procurement and consumption in a holistic manner in Myanmar. This should be inclusive of the private, public, and community sectors and would be vital to improve national AMR surveillance on antibiotic consumption in Myanmar and fluid reporting to WHO.

In conclusion, this operational research study has provided useful insights into antibiotic consumption patterns in Myanmar and could provide impetus towards building a more robust antibiotic consumption surveillance system in the country. Steps are being taken in this direction.

## Figures and Tables

**Table 1:** Numbers and categories of public hospitals included in the study in Myanmar (2014 to 2017).

	Fiscal Year ^1^							
	2014–2015		2015–2016		2016–2017		2017–2018	
	n	(%)	n	(%)	n	(%)	n	(%)
Total hospitals	975		1054		1115		1122	
Study hospitals	325	(33)	338	(32)	346	(31)	347	(31)
<200 beds ^2^	297		310		318		319	
≥200 beds	10		10		10		10	
Central	18		18		18		18	

^1^ Myanmar fiscal year that runs from April to March of each year. ^2^ This category included the same eight regions/states. The budget allocation by regions/states was based on population and not on the number of hospitals. Variations in the number of hospitals did not affect allocated budgets.

**Table 2:** Antibiotic consumption in defined daily dose in public hospitals in Myanmar (2014 to 2017).

Fiscal Year ^1^	Hospitals				Total DDD
	<200 beds		≥200 beds	Central ^3^	
	DDD	DDD/1000/day ^2^	DDD	DDD	DDD
2014–2015	3,601,294	0.6	1,578,391	1,943,167	7,122,852
2015–2016	3,260,830	0.5	1,751,267	1,832,981	6,845,078
2016–2017	1,928,872	0.3	534,712	1,545,929	4,009,513
2017–2018	2,359,850	0.3	2,217,806	1,217,248	5,794,904

DDD = defined daily dose. ^1^ Myanmar fiscal year, which runs from April to March of each year. ^2^ DDD/1000 inhabitants/day. ^3^ The number of beds in central hospital varies between 200 and 1000 beds. Population coverage available only for the region and state procurement (<200 beds).

**Table 3:** Antibiotic consumption in defined daily dose and proportions (%) by pharmacological subgroups (ATC3) in public hospitals in Myanmar (2014 to 2017).

Hospitals	Fiscal year ^1^	Pharmacological Subgroup (ATC3) ^3^							
		Tetracyclines (J01A)	Beta-Lactam and Penicillins (J01C)	Other Beta-Lactam Antibacterials (J01D)	Sulfonamides-Trimethoprim (J01E)	Macrolides, Lincosamides and Streptogamins (J01F)	Aminoglycosides (J01G)	Quinolones (J01M)	Other Antibacterials (J01X) ^2,3^
<200 beds	2014–2015	341,080 (10)	1,115,189 (31)	640,011 (18)	239,871 (7)	247,499 (7)	25,216 (1)	817,478 (23)	174,950 (5)
	2015–2016	143,545 (4)	861,710 (26)	860,029 (26)	95,829 (3)	348,554 (11)	75,981(2)	621,285 (19)	253,897 (8)
	2016–2017	39,650 (2)	560,301 (29)	595,635 (31)	81,150 (4)	213,969 (11)	27,095 (1)	298,645 (16)	112,427 (6)
	2017–2018	59,000 (3)	722,661 (31)	517,036 (22)	36,455 (2)	377,208 (16)	34,114 (1)	363,673 (15)	249,703 (11)
≥200 beds	2014–2015	56,000 (4)	584,873 (37)	312,369 (20)	10,750 (1)	160,925 (10)	25,450 (2)	326,199 (21)	101,825 (7)
	2015–2016	33,050 (2)	1,004,295 (57)	232,598 (13)	28,740 (2)	216,501 (12)	16,748 (1)	142,490 (8)	76,843 (4)
	2016–2017	18,000 (3)	99,418 (19)	190,784 (36)	3550 (1)	86,752 (16)	5593 (1)	107,711(20)	22,903 (4)
	2017–2018	9000 (0)	1,717,691 (78)	249,901 (11)	850 (0)	93,979 (4)	6183 (0)	130,702 (6)	9500 (0.4)
Central	2014–2015	27,000 (1)	448,807 (23)	557,132 (29)	57,456 (3)	179,395 (9)	12,367 (1)	458,260 (24)	202,751 (10)
	2015–2016	79,010 (4)	406,433 (22)	643,577 (35)	16,193 (1)	158,750 (9)	36,071 (2)	347,180 (19)	145,768 (8)
	2016–2017	49,800 (3)	511,177 (33)	537,896 (35)	2075 (0)	116,631(8)	31,182 (2)	237,458 (16)	59,712 (4)
	2017–2018	22,000 (2)	408,771 (34)	392,999 (32)	6325 (1)	150,261 (12)	15,008 (1)	210,995 (17)	10,889 (1)

Data presented in DDD (row %), DDD = defined daily dose. ^1^ Fiscal year refers to Myanmar fiscal year, which is from April to March of each year. ^2^ J01X includes P01AB01 (oral metronidazole). ^3^ ATC3—chemical, pharmacological, or therapeutic subgroup of antibiotic (e.g., J01G is aminoglycosides; No J01B (amphenicols) was recorded).

**Table 4:** Antibiotic consumption in defined daily dose and proportions (%) by Access, Watch, and Reserve (AWaRe) categorization in public hospitals in Myanmar (2014 to 2017).

Hospital	Fiscal Year ^1^	AWaRe Categories			
		Access DDD (%)	Watch DDD (%)	Reserve DDD (%)	Other DDD (%)
<200 beds	2014–2015	1,613,768 (45)	1,459,106 (41)	0	528,419 (15)
	2015–2016	1,233,435 (38)	1,483,655 (46)	0	543,740 (17)
	2016–2017	786,805 (41)	840,763 (44)	0	301,304 (16)
	2017–2018	969,988 (41)	1,064,843 (45)	0	325,019 (14)
≥200 beds	2014–2015	646,544 (41)	765,407 (49)	3100 (0.2)	163,340 (10)
	2015–2016	1,087,374 (62)	550,777(32)	2500 (0.1)	110,616 (6)
	2016–2017	154,036 (29)	334,789 (63)	0	45,886 (9)
	2017–2018	166,5026 (75)	426,146 (19)	0	126,634 (6)
Central	2014–2015	730,985 (38)	1,081,992 (56)	3300 (0.2)	126,890 (7)
	2015–2016	713,071 (39)	1,060,916 (58)	225 (0.01)	58,769 (3)
	2016–2017	684,973 (44)	812,504 (53)	0	48,452 (3)
	2017–2018	465,093 (38)	687,671 (57)	0	64,484 (5)
	2014–2015	2,991,297 (42)	3,306,505 (46)	6400 (0.1)	818,649 (11)
**Total**	2015–2016	3,033,880 (44)	3,095,348 (45)	2725 (0.03)	713,125 (10)
	2016–2017	1,625,814 (41)	1,988,056 (50)	0	395,642 (10)
	2017–2018	3,100,107 (53)	2,178,660 (38)	0	516,137 (9)

DDD = defined daily dose. ^1^ Fiscal year refers to Myanmar fiscal year, which is from April to March of each year.

**Table 5:** Antibiotic consumption in defined daily dose and proportions (%) by route of administration in public hospitals in Myanmar (2014 to 2017).

Hospitals	Route of Administration	Fiscal Year ^1^			
		2014–2015 DDD (%)	2015–2016 DDD (%)	2016–2017 DDD (%)	2017–2018 DDD (%)
<200 beds	Oral	3,116,615 (86)	2,653,428 (81)	1,527,336 (79)	1,911,336 (81)
	Parenteral	484,679 (14)	607,402 (19)	401,536 (21)	448,514 (19)
≥200 beds	Oral	1,199,454 (76)	814,179 (47)	427,406 (80)	665,214 (30)
	Parenteral	378,937 (24)	937,088 (54)	107,306 (20)	1,552,592 (70)
Central	Oral	1,384,785 (71)	1,326,631 (72)	1,049,283 (68)	961,659 (79)
	Parenteral	558,382 (29)	506,350 (28)	496,646 (32)	255,589 (21)

DDD = defined daily dose. ^1^ Fiscal year refers to Myanmar fiscal year, which is from April to March of each year.

**Table 6:** Top ten most consumed oral antibiotics in public hospitals in Myanmar (2014 to 2017).

Hospital					
<200 beds		≥200 beds		Central	
Antibiotic Substance	Proportion	Antibiotic Substance	Proportion	Antibiotic Substance	Proportion
Combinations of penicillins (J01CR50)	16.4	Azithromycin (J01FA10)	15.1	Amoxicillin and enzyme inhibitor (J01CR02)	19.8
Azithromycin (J01FA10)	11.3	Cefixime (J01DD08)	14.0	Cefixime (J01DD08)	19.2
Cefalexin (J01DB01)	10.7	Combinations of penicillins (J01CR50)	11.5	Levofloxacin (J01MA12)	14.4
Cefixime (J01DD08)	10.5	Amoxicillin (J01CA04)	11.4	Azithromycin (J01FA10)	10.4
Ciprofloxacin (J01MA02)	9.5	Amoxicillin and enzyme inhibitor (J01CR02)	7.9	Cefuroxime (J01DC02)	5.2
Amoxicillin (J01CA04)	8.9	Ciprofloxacin (J01MA02)	6.8	Cefalexin (J01DB01)	4.6
Doxycycline (J01AA02)	6.2	Levofloxacin (J01MA12)	6.5	Amoxicillin (J01CA04)	4.5
Sulfamethoxazole and trimethoprim (J01EE01)	4.9	Cefalexin (J01DB01)	4.5	Doxycycline (J01AA02)	3.8
Norfloxacin (J01MA06)	4.7	Metronidazole (P01AB01)	4.3	Ciprofloxacin (J01MA02)	3.7
Amoxicillin and enzyme inhibitor (J01CR02)	3.7	Doxycycline (J01AA02)	3.7	Ofloxacin (J01MA01)	2.3

**Table 7:** Top ten most consumed parenteral antibiotics in a public hospital in Myanmar (2014 to 2017).

Hospital					
<200 beds		≥200 beds		Central	
Antibiotic Substance	Proportion ^1^	Antibiotic Substance	Proportion ^1^	Antibiotic Substance	Proportion ^1^
Metronidazole (J01XD01)	28.0	Amoxicillin and enzyme inhibitor (J01CR02)	72.3	Ceftriaxone (J01DD04)	27.4
Ceftriaxone (J01DD04)	24.1	Procaine benzylpenicillin (J01CE09)	5.8	Amoxicillin and enzyme inhibitor (J01CR02)	15.6
Levofloxacin (J01MA12)	9.8	Ceftriaxone (J01DD04)	5.5	Metronidazole (J01XD01)	15.2
Benzylpenicillin (J01CE01)	6.6	Levofloxacin (J01MA12)	3.4	Levofloxacin (J01MA12)	9.5
Gentamicin (J01GB03)	6.1	Metronidazole (J01XD01)	1.6	Benzylpenicillin (J01CE01)	9.5
Ciprofloxacin (J01MA02)	5.7	Ceftazidime (J01DD02)	1.4	Ceftazidime (J01DD02)	4.3
Combinations of penicillins (J01CR50)	3.6	Amikacin (J01GB06)	1.3	Amikacin (J01GB06)	4.1
Cefotaxime (J01DD01)	3.4	Benzylpenicillin (J01CE01)	1.3	Ceftriaxone and enzyme inhibitor (J01DD63)	3.2
Amoxicillin and enzyme inhibitor (J01CR02)	2.7	Ciprofloxacin (J01MA02)	1.3	Cefoperazone, combinations (J01DD62)	2.6
Ofloxacin (J01MA01)	2.3	Ceftriaxone and enzyme Inhibitor (J01DD63)	0.8	Ciprofloxacin (J01MA02)	1.4

^1^ This is the proportion on 100% of all antibiotics consumed.

**Table 8:** Consumption of other beta-lactam antibacterials (J01D) in defined daily dose and proportions (%) in public hospitals in Myanmar (2014 to 2017).

Hospital	Fiscal Year ^1^	Antibiotic Ubstance				
		First-Generation Cephalosporins (J01DB)	Second-Generation Cephalosporins (J01DC)	Third-Generation Cephalosporins (J01DD)	Fourth-Generation Cephalosporins (J01DE)	Carbapenems (J01DH)
<200 beds	2014–2015	240,263 (38)	23,400 (4)	374,973 (57)	1375 (0.2)	0
	2015–2016	312,984 (36)	6070 (0.7)	540,290 (63)	685 (0.1)	0
	2016–2017	253,345 (43)	13,730 (2)	328,485 (55)	75 (0)	0
	2017–2018	188,174 (36)	13,135 (3)	313,978 (61)	1750 (0.3)	0
≥200 beds	2014–2015	34,138 (11)	42,534 (14)	221,532 (71)	7565 (2)	6600 (2)
	2015–2016	32,604 (14)	38,925 (17)	152,570 (66)	6000 (3)	2500 (1)
	2016–2017	37,850 (20)	778 (0.4)	150,479 (79)	1599 (1)	78 (0)
	2017–2018	36,313 (15)	21,400 (9)	191,146 (77)	963 (0.4)	79 (0)
Central	2014–2015	80,583 (15)	91,790 (17)	370,926 (67)	6040 (1)	7794 (1)
	2015–2016	73,448 (11)	117,319 (18)	446,824 (69)	3666 (0.6)	2321 (0.5)
	2016–2017	47,015 (9)	26,268 (5)	457,160 (85)	2545 (0.5)	4908 (0.9)
	2017–2018	24,365 (6)	28,877 (7)	335,071 (85)	1145 (0.3)	3541 (0.9)

^1^ Fiscal year refers to Myanmar fiscal year, which is from April to March of each year.

## Data Availability

The data presented in this study are available on request from the corresponding author.

## Open access statement and disclaimer

In accordance with WHO’s open-access publication policy for all work funded by WHO or authored/co-authored by WHO staff members, the WHO retains the copyright of this publication through a Creative Commons Attribution IGO License (http://creativecommons.org/licenses/by/3.0/igo/legalcode; accessed on 7 April 2021), which permits unrestricted use, distribution, and reproduction in any medium provided the original work is properly cited. In any use of this article, there should be no suggestion that WHO endorses any specific organization, products, or services. The views expressed in this article are those of the authors and do not necessarily reflect those of their affiliated institutions. The use of the WHO logo is not permitted. This notice should be preserved along with the article’s original URL.
